# The association between systemic immune-inflammation index and periodontitis in adults with and without hyperlipidemia: a population-based study

**DOI:** 10.1038/s41405-026-00422-3

**Published:** 2026-04-01

**Authors:** Ying Xie, Shuangying Zhou

**Affiliations:** Department of Periodontology, Peking University School and Hospital of Stomatology & National Center for Stomatology & National Clinical Research Center for Oral Diseases & National Engineering Research Center of Oral Biomaterials and Digital Medical Devices, Beijing, China

**Keywords:** Oral pathology, Periodontitis

## Abstract

**Purpose:**

To investigate the association between SII and periodontitis, and to determine whether this association differs by hyperlipidemia status in adults.

**Methods:**

21,283 U.S. adults aged ≥18 years were enrolled from NHANES 1999–2014. Multivariable logistic regression models were employed to assess the association between SII and periodontitis while controlling for potential confounding factors. We further explored non-linear dose-response relationships using two-piecewise regression model to identify any threshold effect of SII on periodontitis risk. Robustness of the findings was evaluated through sensitivity analyses.

**Results:**

Periodontitis was prevalent in 6,758 individuals (31.75%). In stratified analyses, this pattern was evident among individuals with hyperlipidemia: we identified an inflection point at SII ≈ 522. Within the lower SII range, a 500-unit increment demonstrated an inverse association with periodontitis susceptibility, yielding a significant 18% risk reduction (OR = 0.82, 95% CI 0.68–0.99, *P* = 0.036). Conversely, in the upper SII spectrum, identical 500-unit increments corresponded to a substantial 15% heightened periodontitis risk (OR = 1.15, 95% CI 1.05–1.25, *P* = 0.002). A significant non-linear dose–response was found in hyperlipidemic individuals (*P* for threshold effect = 0.005). In contrast, among individuals without hyperlipidemia, no significant association between SII and periodontitis was detected. Sensitivity analyses, including the exclusion of participants with malignancies, stratification by sex and using alternative periodontitis definitions, yielded consistent results, reinforcing the robustness of our findings.

**Conclusions:**

SII demonstrated a non-linear association with periodontitis that was restricted to individuals with hyperlipidemia. In hyperlipidemic adults, moderate increases in SII (within a lower range) were associated with reduced odds of periodontitis, whereas an SII beyond ~522 was significantly associated with higher disease prevalence.

## Introduction

Periodontitis is an inflammatory condition affecting the periodontium, characterized by gingival inflammation, as well as destruction of alveolar bone. Initiated by complex microbiota, it is further exacerbated by the host’s immune-inflammatory response [1, 2]. Beyond oral health, periodontitis signals a broader medical concern, affecting patient quality of life and potentially interconnecting with diverse systemic disease mechanisms [3–6].

Accumulating evidence suggests a bidirectional relationship between periodontitis and various chronic systemic conditions, including cardiovascular diseases and metabolic disorders. Notably, hyperlipidemia—a common metabolic condition defined by lipid dysregulation—has been correlated with increased periodontal disease risk [7–9]. Epidemiological studies indicate that individuals with adverse lipid profiles, such as low high-density lipoprotein (HDL) cholesterol, elevated triglycerides, or high low-density lipoprotein (LDL) cholesterol, are predisposed to develop periodontitis in comparison to individuals with normal lipid levels [10, 11]. Conversely, severe periodontitis can contribute to systemic inflammation that disrupts lipid metabolism, potentially exacerbating hyperlipidemia [12]. Given this bidirectional relationship, patients with hyperlipidemia may experience an enhanced inflammatory state that adversely affects their periodontal health.

Chronic low-grade systemic inflammation is a critical characteristic shared by both periodontitis and metabolic disorders. The Systemic Immune-Inflammation Index (SII), calculated by multiplying peripheral blood neutrophil and platelet counts, subsequently divided by lymphocyte count, is increasingly recognized as a prognostic indicator for chronic inflammatory conditions [13, 14]. Elevated SII has been linked to increased severity of hypertension and dyslipidemia, suggesting its potential to reflect systemic inflammatory burden in metabolic conditions [15, 16].

Recent researches have elucidated the role of SII in periodontal disease. A hospital-based case-control study found significantly increased SII in patients with severe generalized periodontitis, demonstrating a robust association between elevated SII and higher odds of severe periodontitis [17]. Additionally, a cross-sectional analysis of U.S. adults identified a J-shaped relationship between SII and periodontitis prevalence, indicating a potentially non-linear interaction influenced by host factors [18]. Nevertheless, previous research has not thoroughly explored how this relationship is modulated by systemic conditions. Given the established connections between periodontitis and metabolic disorders, we propose that the association between SII (as an indicator of systemic inflammation) and periodontitis may be exacerbated in individuals with hyperlipidemia or other metabolic risk factors.

In this context, employing the National Health and Nutrition Examination Survey (NHANES) dataset covering 1999–2014, our investigation specifically focused on: (1) evaluate the dose-response association between SII and the prevalence of periodontitis by hyperlipidemia status, and (2) test for a potential threshold or non-linear effect in this association.

## Methods

### Study population

**Fig. 1:**
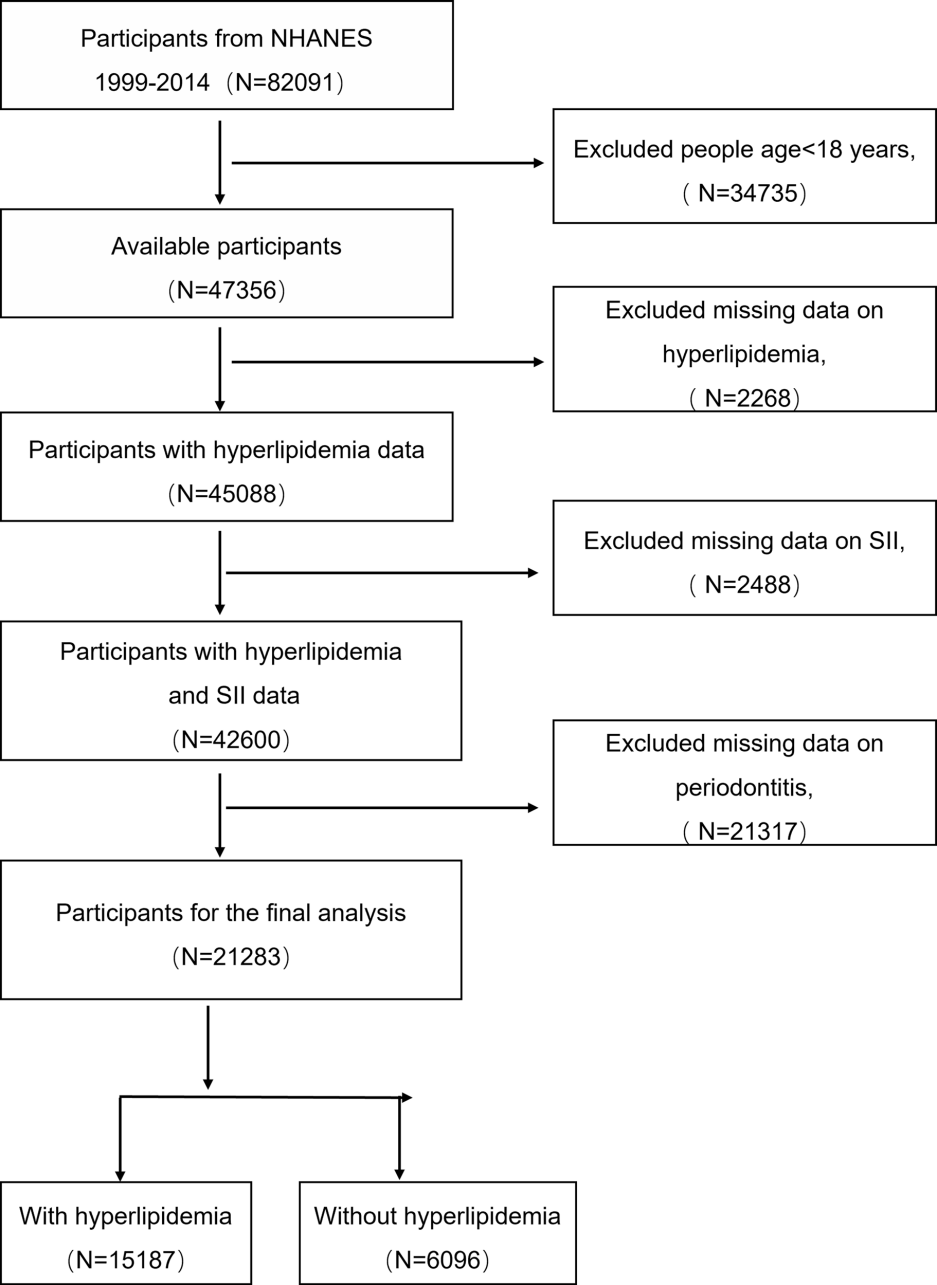
Study flowchart.

NHANES, a cross-sectional study, is conducted by the U.S. National Center for Health Statistics to produce nationally representative data on the health and nutrition of the non-institutionalized civilian. Our study utilizes data from the NHANES covering 1999–2014. From the initial cohort of 82,091 participants, several exclusion criteria were applied: individuals aged under 18 years (*N* = 34,735) were excluded, as the study focused on adults; participants lacking information on hyperlipidemia (*N* = 2268), SII (*N* = 2488), and periodontitis (*N* = 21,317) were removed (Fig. 1). Ultimately, the final analytical sample included 21,283 participants. All participants provided informed consent, and the protocol was approved by the NCHS Research Ethics Review Board. This study follows the strengthening the reporting of observational studies in epidemiology (STROBE) guidelines [19].

### Periodontitis assessment

The outcome of interest, periodontitis, was determined through a standardized periodontal examination conducted by trained dental examiners. In NHANES 1999–2004, a half-mouth examination protocol was used (measuring probing depth and attachment loss on select index teeth in two quadrants), whereas from 2009 onward, a full-mouth periodontal examination (FMPE) (excluding third molars) was implemented. To ensure comparability across survey cycles, we applied the established CDC/AAP criteria for diagnosing periodontitis (Supplementary Table 1) [18]. Participants were classified as periodontitis group encompassing mild, moderate, or severe periodontitis; those not meeting any of these thresholds were classified as periodontally healthy/without periodontitis.

### SII (exposure)

The primary exposure SII was calculated from a complete blood count with differential, which was measured as part of NHANES laboratory assessments. The SII refers to: (neutrophils count × platelets count)/lymphocytes count. All counts were measured on automated hematology analyzers.

### Effect modifier—hyperlipidemia definition

Given our interest in hyperlipidemia as an effect modifier, we defined the hyperlipidemia status for each participant based on a combination of laboratory data and self-reported information. Hyperlipidemia is defined in accordance with the NCEP ATP III and ACC/AHA guidelines, relying on the following laboratory parameters obtained from venous blood samples: triglyceride levels ≥150 mg/dL, total cholesterol ≥200 mg/dL, HDL levels ≤40 mg/dL for men and ≤50 mg/dL for women, and LDL levels ≥130 mg/dL [20]. Participants were identified as having hyperlipidemia if they answered “yes” to ever being informed by a physician about high cholesterol, or were currently taking cholesterol-lowering medications [21, 22].

### Covariates

We included a wide range of covariates, such as age, sex, race/ ethnicity, marital status, and Poverty-to-income ratio (PIR) in our analyses, selected based on prior knowledge of risk factors for periodontitis and potential confounders of the SII–periodontitis relationship. PIR is categorized into five distinct groups to evaluate economic status: “Poor” (< 1.00); “Nearly Poor” (1.00–1.99); “Middle Income” (2.00–4.00); “High Income” (≥ 4.00); and unknown (not acquired). We also included educational attainment as an additional socio-economic indicator.

Cigarette smoking status (never, former, or current smoker) was included, considering the well-known strong impact of smoking on periodontitis risk. Alcohol use was included as a covariate, classified as: nondrinker, moderate drinker, or heavy drinker (daily intake of over 1 drink for women or over 2 drinks for men on average). We also adjusted for total physical activity, computed as weekly metabolic equivalent (MET) minutes, and categorized this variable into tertiles (low, medium, high) for analysis.

Key health status covariates included hypertension [23] and diabetes mellitus [24]. These conditions are relevant because they are associated with systemic inflammation and have known links to periodontal disease. Inclusion of hypertension and diabetes in the models helps account for general health status differences that might confound the inflammation–periodontitis association.

### Statistical analysis

Continuous variables were presented as mean ± SD; categorical variables as proportions. Multivariable logistic regression was employed to assess the relationship between SII and periodontitis risk. Our Model was fully adjusted for basic demographics (age, sex, and race), health behaviors (smoking, alcohol, physical activity), socioeconomic factors (marital status, PIR, education), BMI, hypertension, and diabetes status. We also evaluated SII in tertiles to examine its association with periodontitis and plotted the prevalence of periodontitis across SII tertiles. Dummy variables were used to indicate missing covariate values, including education, PIR, marital status, smoking status, alcohol use, and total physical activity.

Given evidence from prior studies of non-linearity, we formally tested for a non-linear relationship between SII and periodontitis using restricted cubic spline functions in the logistic model [18]. To further probe this, we employed a piecewise (two-segment) logistic regression approach. The turning point for the SII was determined using “exploratory” analyses, which is to move the trial turning point along the pre-defined interval and pick up the one which gave maximum model likelihood. We also performed a log-likelihood ratio test and compared the one-line linear regression model with the two-piecewise linear regression model. We used the bootstrap resampling method to calculate the 95% CI for the turning point, as described in the previous analysis [25]. Crucially, we examined effect modification by hyperlipidemia. We found that the association between the SII and periodontitis varies according to the hyperlipidemia status. Interestingly, among those with hyperlipidemia, a clear threshold was confirmed (~522, as mentioned), whereas no meaningful threshold was evident for those without hyperlipidemia. Thus, in the hyperlipidemia stratum, we report results for SII ≥ 522 and SII < 522, while in the non-hyperlipidemia stratum, we report the overall association (which was null).

Finally, we performed several sensitivity analyses: (1) excluding individuals with malignancies; (2) stratifying analyses by sex; (3) using alternative periodontitis definitions (e.g., categorizing no/mild cases as the outcome = 0 and moderate/severe periodontitis as the outcome = 1); (4) defining hyperlipidemia based solely on laboratory criteria, regardless of treatment status. In each sensitivity analysis, the results did not materially change, and we report key comparisons in the Results section.

All tests were two-tailed with a significance level of *α* = 0.05. Statistical analyses were performed using the statistical packages R (R Foundation for Statistical Computing, http://www.r-project.org) and Empower (R) (X&Y Solutions, Inc.; www.empowerstats.com).

## Results

### Participant characteristics

The characteristics of 21,283 participants were systematically distributed across the SII tertiles (T1: 11.88–391.68, T2: 391.69–605.08, T3: 605.13–2500) (Table 1). Significant differences were observed in SII, with means of 286.21 ± 73.46 for T1, 490.37 ± 61.12 for T2, and 911.82 ± 513.05 for T3 (*P* < 0.001). Participants in T1 were older (mean age 48.02 ± 16.73 years), had a greater proportion of males (54.92%), Non-Hispanic Blacks (29.25%), and current smokers (17.54%), while T3 participants were younger (mean age 45.76 ± 18.41 years) with a greater proportion of females (58.03%), Non-Hispanic Whites (51.21%), and heavy alcohol drinkers (18.37%). Additionally, BMI, platelet and neutrophil counts, and the prevalence of hyperlipidemia and periodontitis differed significantly across tertiles (*P* < 0.001 for all), suggesting potential associations between SII and these demographic, lifestyle, and clinical factors.

**Table 1:** Characteristics of the participants (*N* = 21,283).

Characteristics	SII				*P*-value
	Overall	T1	T2	T3	
	(*n* = 21,283)	(*n* = 7094)	(*n* = 7094)	(*n* = 7095)	
SII range	11.88–2500	11.88–391.68	391.69–605.08	605.13–2500	
SII, mean ± SD	562.81 ± 398.31	286.21 ± 73.64	490.37 ± 61.12	911.82 ± 513.05	<0.001
Age	46.87 ± 17.46	48.02 ± 16.73	46.83 ± 17.12	45.76 ± 18.41	<0.001
Sex					<0.001
Male	10432 (49.02%)	3896 (54.92%)	3558 (50.16%)	2978 (41.97%)	
Female	10851 (50.98%)	3198 (45.08%)	3536 (49.84%)	4117 (58.03%)	
Race					<0.001
Non-Hispanic White	9546 (44.85%)	2590 (36.51%)	3323 (46.84%)	3633 (51.21%)	
Mexican American	4335 (20.37%)	1277 (18.00%)	1488 (20.98%)	1570 (22.13%)	
Non-Hispanic Black	4215 (19.80%)	2075 (29.25%)	1223 (17.24%)	917 (12.92%)	
Other Hispanic	1547 (7.27%)	514 (7.25%)	542 (7.64%)	491 (6.92%)	
Others	1640 (7.71%)	638 (8.99%)	518 (7.30%)	484 (6.82%)	
Total physical activity^a^ (MET/week)					<0.001
<600	6588 (30.95%)	1965 (27.70%)	2257 (31.82%)	2366 (33.35%)	
>=600	9209 (43.27%)	3378 (47.62%)	3113 (43.88%)	2718 (38.31%)	
BMI^a^ (kg/m^2^)	28.60 ± 6.54	28.13 ± 6.08	28.63 ± 6.31	29.03 ± 7.13	<0.001
Education^a^, *n* (%)					0.002
< High school	2352 (11.05%)	854 (12.04%)	774 (10.91%)	724 (10.20%)	
High school	8381 (39.38%)	2693 (37.96%)	2777 (39.15%)	2911 (41.03%)	
å High school	10527 (49.46%)	3538 (49.87%)	3536 (49.84%)	3453 (48.67%)	
PIR^a^, mean ± SD	2.63 ± 1.65	2.65 ± 1.65	2.65 ± 1.66	2.57 ± 1.64	0.008
PIR^a^, *n* (%)					0.084
Poor	3845 (18.07%)	1223 (17.24%)	1291 (18.20%)	1331 (18.76%)	
Nearly poor	4896 (23.00%)	1647 (23.22%)	1603 (22.60%)	1646 (23.20%)	
Middle income	5281 (24.81%)	1750 (24.67%)	1746 (24.61%)	1785 (25.16%)	
High income	5524 (25.95%)	1871 (26.37%)	1897 (26.74%)	1756 (24.75%)	
Marital status^a^, *n* (%)					<0.001
Married/Living with Partner	12774 (60.02%)	4381 (61.76%)	4293 (60.52%)	4100 (57.79%)	
Widowed/Divorced/Separated	4047 (19.02%)	1326 (18.69%)	1349 (19.02%)	1372 (19.34%)	
Never married	4101 (19.27%)	1277 (18.00%)	1333 (18.79%)	1491 (21.01%)	
Smoke status^a^, *n* (%)					<0.001
Never	10944 (51.42%)	3792 (53.45%)	3671 (51.75%)	3481 (49.06%)	
Former	4875 (22.91%)	1647 (23.22%)	1602 (22.58%)	1626 (22.92%)	
Now	4028 (18.93%)	1244 (17.54%)	1339 (18.88%)	1445 (20.37%)	
Alcohol consumption^a^, *n* (%)					<0.001
Never	2615 (12.29%)	931 (13.12%)	832 (11.73%)	852 (12.01%)	
Former	3264 (15.34%)	1082 (15.25%)	1001 (14.11%)	1181 (16.65%)	
Mild	6444 (30.28%)	2245 (31.65%)	2222 (31.32%)	1977 (27.86%)	
Moderate	2781 (13.07%)	933 (13.15%)	939 (13.24%)	909 (12.81%)	
Heavy	3653 (17.16%)	1123 (15.83%)	1227 (17.30%)	1303 (18.37%)	
Platelet (10^3^ cells/μL), mean ± SD	253.17 ± 66.54	216.96 ± 51.52	252.21 ± 54.30	290.32 ± 70.62	<0.001
Lymphocyte (10^3^ cells/μL), mean ± SD	2.12 ± 1.07	2.37 ± 1.59	2.11 ± 0.64	1.88 ± 0.60	<0.001
Neutrophils (10^3^ cells/μL), mean ± SD	4.30 ± 1.86	3.05 ± 0.98	4.13 ± 1.12	5.72 ± 2.15	<0.001
Hypertension^a^, *n* (%)					0.12
No	13378 (62.87%)	4405 (62.09%)	4521 (63.76%)	4452 (62.77%)	
Yes	7900 (37.13%)	2689 (37.91%)	2570 (36.24%)	2641 (37.23%)	
Diabetes Mellitus^a^, *n* (%)					0.164
No	16421 (79.88%)	5623 (79.57%)	5633 (80.64%)	5165 (79.41%)	
Diabetes mellitus	2951 (14.36%)	1028 (14.55%)	956 (13.69%)	967 (14.87%)	
IFG	730 (3.55%)	241 (3.41%)	258 (3.69%)	231 (3.55%)	
IGT	454 (2.21%)	175 (2.48%)	138 (1.98%)	141 (2.17%)	
Hyperlipidemia					<0.001
No	6096 (28.64%)	2160 (30.45%)	2005 (28.26%)	1931 (27.22%)	
Yes	15187 (71.36%)	4934 (69.55%)	5089 (71.74%)	5164 (72.78%)	
Periodontitis					<0.001
No	14525 (68.25%)	4595 (64.77%)	4919 (69.34%)	5011 (70.63%)	
Yes	6758 (31.75%)	2499 (35.23%)	2175 (30.66%)	2084 (29.37%)	

Missing values: BMI (*n* = 186, 0.88%), education (*n* = 23, 0.11%), PIR (*n* = 1737, 8.16%), marital status (*n* = 361, 1.70%), smoke status (*n* = 1436, 6.75%), alcohol use (*n* = 2526, 11.87%), total physical activity (*n* = 5486, 25.78%), Diabetes Mellitus (*n* = 727, 3.42%) and hypertension (*n* = 5, 0.023%).

*IFG* impaired fasting glycaemia, *IGT* impaired glucose tolerance.

### The univariate analysis of factors associated with periodontitis

Table 2 revealed significant findings across demographic, socioeconomic, and health-related variables. Age was positively correlated with periodontitis (OR: 1.05, *P* < 0.0001). Females had lower odds compared to males (OR: 0.55, *P* < 0.0001). Ethnic variations showed Non-Hispanic Blacks faced higher odds (OR: 1.81, *P* < 0.0001), while Individuals with lower educational attainment and those living in high-poverty conditions also exhibited higher likelihoods of the disease. Smoking increased risks significantly, with current smokers showing an OR of 1.93 (*P* < 0.0001). Interestingly, regular physical activity (≥600 MET/week) correlated with higher odds (OR: 2.46, *P* < 0.0001). Additionally, the presence of hypertension, diabetes, and hyperlipidemia greatly increased odds. Higher BMI was also associated with raised risk levels.

**Table 2:** Univariate analysis for periodontitis.

Covariate	Statistics	OR (95%CI)	*P*-value
Age	46.87 ± 17.46	1.05 (1.04, 1.05)	<0.0001
Sex			
Male	10432 (49.02%)	Reference	
Female	10851 (50.98%)	0.55 (0.52, 0.58)	<0.0001
Race			
Non-Hispanic White	9546 (44.85%)	Reference	
Mexican American	4335 (20.37%)	1.31 (1.21, 1.41)	<0.0001
Non-Hispanic Black	4215 (19.80%)	1.81 (1.67, 1.95)	<0.0001
Other Hispanic	1547 (7.27%)	1.89 (1.69, 2.11)	<0.0001
Others	1640 (7.71%)	1.90 (1.70, 2.11)	<0.0001
Total physical activity (MET/week)			
<600	6588 (41.70%)	Reference	
>=600	9209 (58.30%)	2.46 (2.28, 2.65)	<0.0001
BMI (kg/m2)	28.60 ± 6.54	1.02 (1.02, 1.03)	<0.0001
Education level			
< high school	2352 (11.06%)	Reference	
High school	8381 (39.42%)	0.60 (0.55, 0.66)	<0.0001
>Above high school	10527 (49.52%)	0.40 (0.37, 0.44)	<0.0001
PIR			
Poor	3845 (18.07%)	Reference	
Nearly poor	4896 (23.00%)	1.00 (0.91, 1.09)	0.9435
Middle income	5281 (24.81%)	0.71 (0.65, 0.77)	<0.0001
High income	5524 (25.95%)	0.44 (0.40, 0.48)	<0.0001
Missing	1737 (8.16%)	0.88 (0.78, 0.99)	0.0343
Marital status			
Married/Living with Partner	12774 (61.06%)	Reference	
Widowed/Divorced/Separated	4047 (19.34%)	1.64 (1.53, 1.76)	<0.0001
Never married	4101 (19.60%)	0.46 (0.42, 0.50)	<0.0001
Smoke status			
Never	10944 (51.42%)	Reference	
Former	4875 (22.91%)	1.57 (1.46, 1.68)	<0.0001
Now	4028 (18.93%)	1.93 (1.79, 2.08)	<0.0001
Missing	1436 (6.75%)	0.07 (0.05, 0.10)	<0.0001
Alcohol consumption			
Never	2615 (12.29%)	Reference	
Heavy	3653 (17.16%)	1.01 (0.91, 1.12)	0.8511
Moderate	2781 (13.07%)	0.69 (0.61, 0.77)	<0.0001
Mild	6444 (30.28%)	0.87 (0.79, 0.96)	0.006
Former	3264 (15.34%)	1.38 (1.24, 1.53)	<0.0001
Missing	2526 (11.87%)	0.42 (0.37, 0.47)	<0.0001
Hypertension			
No	13378 (62.87%)	Reference	
Yes	7900 (37.13%)	2.36 (2.22, 2.50)	<0.0001
Diabetes Mellitus			
No	16421 (79.88%)	Reference	
Diabetes Mellitus	2951 (14.36%)	2.91 (2.68, 3.15)	<0.0001
IFG	730 (3.55%)	1.93 (1.66, 2.25)	<0.0001
IGT	454 (2.21%)	3.02 (2.50, 3.64)	<0.0001
Hyperlipidemia			
No	6096 (28.64%)	Reference	
Yes	15187 (71.36%)	1.48 (1.38, 1.58)	<0.0001

*OR* odds ratio.

### Association between SII and periodontitis in hyperlipidemia

**Fig. 2:**
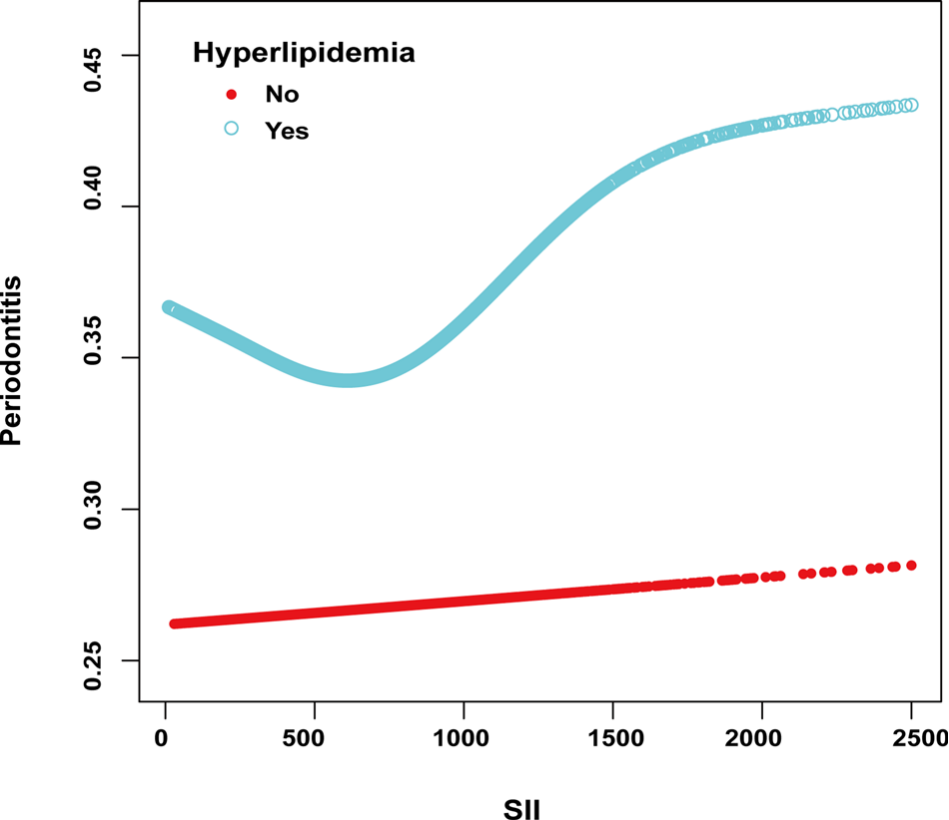
Relationship between SII and periodontitis stratified by hyperlipidemia in fully adjusted models.

Considering our primary interest in effect modification, we examined the association between the SII and the risk of periodontitis, stratified by hyperlipidemia status. Figure 2 illustrates this relationship, showing a non-linear association between SII values and periodontitis risk among individuals with hyperlipidemia, with a *P*-value for non-linearity of 0.005. Conversely, in individuals without hyperlipidemia, the risk of periodontitis remained stable across varying SII levels, suggesting that the SII significantly influences periodontitis risk only in the presence of hyperlipidemia.

Table 3 presents the threshold effect analyses of the SII on the risk of periodontitis, further elucidating the stratified results. In the hyperlipidemia subgroup, variations in the SII index demonstrated a notable association with periodontitis risk. In fully adjusted logistic regression, for SII above 522, each additional 500 units was associated with 15% higher odds of periodontitis (OR = 1.15, 95% CI 1.05–1.25), highlighting that elevated SII levels correlate with an elevated risk of periodontitis in hyperlipidemic individuals. In contrast, those with an SII less than 522 exhibited a reduced odds of periodontitis (OR: 0.82, 95% CI: 0.68–0.99). Among normolipidemic participants, SII showed no significant correlation with periodontitis risk.

**Table 3:** Threshold effect analyses of SII (per 500 units increase) on risk of periodontitis in hyperlipidemia and non-hyperlipidemia in fully adjusted models.

SII index	OR (95%CI)	*P*-value
hyperlipidemia		
Model I		
One line effect, SII per 500 units increase	1.05 (0.99, 1.12)	0.122
Model II		
Turning point (K)	SII index=522	
SII index <522, SII per 500 units increase	0.82 (0.68, 0.99)	0.036
SII index ≥ 522, SII per 500 units increase	1.15 (1.05, 1.25)	0.002
*P* value for LRT test*		0.011
non-hyperlipidemia		
SII per 500 units increase	1.02 (0.91, 1.13)	0.784

* *P* < 0.05 indicates that Model II is significantly different from Model I. Adjusted for age, sex, race, smoke status, alcohol consumption, physical activity, marital status, PIR, education, BMI, hypertension, and diabetes status.

*OR* odds ratio, *CI* Confidence Interval, *LRT* Logarithm Likelihood Ratio Test.

In addition, SII was analyzed in tertiles to further assess its association with periodontitis(Supplementary Table 2).

### Sensitivity analyses

In addition, various sensitivity analyses were conducted, including removing participants with malignancies, stratification by sex, and the use of alternative definitions of periodontitis. To further address potential confounding related to medication, an additional sensitivity analysis was conducted wherein hyperlipidemia was defined strictly by objective laboratory criteria, irrespective of lipid-lowering treatment status. The main results were robust across the analyses (Supplementary Figs. 1 and 2). The consistency across these checks strengthens confidence in the validity of our results.

## Discussion

In this study, we found that the relationship between the SII and periodontitis is highly dependent on the presence of hyperlipidemia. Our key finding indicates that SII is significantly associated with periodontal disease exclusively in individuals with hyperlipidemia. In the hyperlipidemic subgroup, we observed a clear J-shaped association: both relatively low and very high SII values were associated with greater odds of periodontitis, with an intermediate SII ~522 associated with the lowest risk. In contrast, among individuals without hyperlipidemia, SII exhibited no significant association with periodontitis. Our research provides the initial evidence of dose–response relationship between SII and periodontitis in hyperlipidemia adults. These findings extend prior research on systemic inflammation and periodontal disease and suggest that metabolic status (dyslipidemia) plays a crucial role in linking systemic immune profiles to oral health outcomes.

Our results in the hyperlipidemia subgroup align with and build upon the work of Cao et al., who reported a J-shaped association between SII and periodontitis, with an inflection point at log2(SII) = 8.66 (approximately SII ≈ 400) in the general adult population using NHANES 2009–2014 [18]. We found a similar J-shaped dose–response relationship in hyperlipidemic individuals, although our analysis indicated an inflection point around 522, compared to 400 in Cao et al.’s study. This discrepancy may stem from different analytic methodologies or the inclusion of additional NHANES cycles in our research. While the Cao et al. study did not explicitly focus on hyperlipidemia, it highlighted racial differences, noting that the protective association of low SII was most prominent in White individuals. Our findings imply that metabolic health may be crucial to this non-linear relationship. Similarly, Guo et al. reported that SII was linked to periodontitis predominantly in individuals over 50 years old, aligning with our results in the hyperlipidemic subgroup [26]. The increased prevalence of hyperlipidemia and other inflammatory conditions in older adults may explain their findings. Both studies emphasize that in a pro-inflammatory or high-risk host environment, SII serves as a significant marker of periodontal disease risk, while its influence diminishes in healthier populations. In patients with hyperlipidemia, lipid-induced chronic low-grade inflammation (“meta-inflammation”) synergistically elevates conventional markers like hs-CRP and total leukocyte counts. However, by uniquely integrating platelet counts with innate and adaptive immune cells, the SII provides a comprehensive reflection of the systemic “immunothrombotic” burden. This integration effectively captures the compounding inflammatory and pro-coagulant pathways shared by both dyslipidemia and severe periodontitis [27]. Our observation that SII’s effect is pronounced in the context of hyperlipidemia may reconcile mixed results in the existing literature. It underscores the notion that the impact of systemic inflammation on periodontal disease is context-dependent; a high SII in a healthy individual may not correlate with periodontal breakdown, whereas a similar level of SII in someone with metabolic disruptions could signal underlying or imminent periodontitis.

The SII comprehensively reflects systemic immune-inflammatory status based on circulating neutrophil, platelet, and lymphocyte counts [28, 29]. In our study, the relationship between the SII and severe periodontitis followed a J-shaped curve, underscoring the critical need for immune homeostasis. The inflection point (SII ≈ 522) represents an optimal immunological balance, effectively containing dysbiotic biofilms without inflicting collateral tissue injury. Interestingly, the left arm (low SII) indicates an elevated periodontitis risk. While this may partially stem from an initial inadequate innate response—where impaired neutrophil function fails to control bacterial plaque [30, 31]—we postulate it primarily reflects a chronic adaptive shift. According to the classic Page and Schroeder model, advanced periodontal lesions transition from an innate, neutrophil-dominated infiltrate to an adaptive, lymphocyte- and plasma cell-dominated environment [32]. Thus, a diminished SII (driven by elevated systemic lymphocytes) may essentially capture this chronic, destructive phase of periodontitis. Conversely, the right arm (high SII) signifies an exaggerated inflammatory phenotype exacerbated by hyperlipidemia [33–36]. In this state, altered lipid metabolism primes pro-inflammatory neutrophils to induce tissue destruction via excessive reactive oxygen species (ROS) and proteolytic enzymes (e.g., MMPs) [37, 38]. This destruction is further amplified by platelet-driven microthrombi [39–41] and diminished lymphocyte-mediated regulation [42–44].

Moreover, the observed associations likely reflect a complex reciprocal interaction rather than a unidirectional causal effect. Severe periodontitis constitutes a chronic infectious burden, allowing periodontal pathogens and their products (e.g., lipopolysaccharide) to enter the circulation and trigger systemic inflammatory responses, thereby secondarily elevating indices like the SII. Ultimately, the SII may serve as a critical barometer reflecting the delicate equilibrium between protective immunity and destructive inflammation. Future prospective cohort studies are warranted to longitudinally track the SII in hyperlipidemic populations to validate these hypotheses.

In participants without hyperlipidemia, the lack of association between SII and periodontitis risk suggests their systemic inflammation is less impactful on periodontal disease. In metabolically healthy individuals, local factors—such as plaque burden, hygiene, and specific pathogens—may more significantly influence periodontitis risk than minor systemic immune variations. Furthermore, hyperlipidemia may alter immune cell function, underscoring SII’s diagnostic and prognostic significance in pathological assessment.

This study underscores the multifactorial nature of periodontitis, highlighting how systemic conditions such as dyslipidemia can alter the impact of systemic inflammatory markers on disease risk. To enhance the translational impact of our findings, it is essential to contextualize the clinical relevance of the SII from both clinical and public health perspectives. First, the SII emerges as a valuable risk indicator for periodontitis, particularly among patients with hyperlipidemia. Based on the observed J-shaped curve, an optimal immune-inflammatory balance in this population corresponds to an SII level of ~522. Extreme deviations from this nadir—such as an SII < 200 or > 1000—could serve as practical thresholds prompting a dental referral. Mechanistically, an extremely low SII may indicate potential immune insufficiency, while a markedly high SII suggests an overactive inflammatory state. Although the magnitude of risk associated with an elevated SII (OR = 1.15 per 500-unit increase above the threshold) is more modest than established behavioral risk factors like active smoking (OR = 1.93), the risk is continuous and cumulative. Therefore, hyperlipidemic patients with extreme SII deviations could benefit from thorough periodontal examinations and early interventions. Second, our results reinforce the importance of a holistic, systemic approach to oral health. Managing hyperlipidemia through dietary changes, lifestyle modifications, or medications may yield benefits beyond cardiovascular health, potentially enhancing periodontal outcomes by modulating systemic inflammation. Ultimately, close clinical attention should be paid to the SII in hyperlipidemic patients.

Nevertheless, our study has some inherent methodological constraints. The cross-sectional study does not allow for the establishment of temporal causality; we cannot determine whether high SII causes periodontitis, vice versa, or if both are influenced by a shared underlying factor. It is plausible that severe periodontitis itself elevates systemic inflammatory markers, such as WBC counts, thereby raising SII. Thus, the association observed in hyperlipidemic individuals may reflect a stronger systemic inflammatory response to periodontitis. Longitudinal studies are needed to assess whether baseline SII predicts future periodontal breakdown across various subgroups. Additionally, potential residual confounding exists; while we adjusted for many factors, diet was not explicitly included (though partly reflected in hyperlipidemia), and unmeasured variables like genetic susceptibility or oral hygiene practices could influence results. Because poor oral hygiene and pro-inflammatory (e.g., high-sugar) diets are well-established shared risk factors that simultaneously exacerbate both systemic inflammation and periodontal tissue destruction, they act as positive confounders. Consequently, the omission of these variables likely introduces a positive bias (bias away from the null). Although our dataset contained missing values for some variables, we utilized advanced statistical techniques to handle these missing data, thereby minimizing potential bias. Furthermore, earlier cycles only provided partial data of periodontal status, potentially leading to misclassification of mild cases. This may bias the associations towards the null, making it harder to detect links. Another limitation is our definition of hyperlipidemia by combining laboratory parameters with self-reported information. Although guideline-based, this approach may introduce misclassification bias due to reliance on a single lipid measurement and on patient recall. Notably, exposure misclassification from these errors would bias the results toward the null, potentially underestimating the association between SII and periodontitis in adults with and without hyperlipidemia. Lastly, we lacked data on certain systemic inflammatory diseases, including rheumatoid arthritis or chronic infections, that might influence SII and periodontitis. However, these conditions have low prevalence in the population.

### Ethics approval and consent to participate

Ethical review and approval were waived for this study because no additional institutional review board approval was required for the secondary analysis. All authors have reviewed and approved the manuscript for publication.

## Conclusion

In summary, the SII is non-linearly associated with periodontitis, and importantly, this association is contingent on hyperlipidemia status. In hyperlipidemic adults, a J-shaped association between SII and periodontitis is observed: both hypo-immune (low SII) and hyper-inflammatory (high SII) states are linked to increased periodontitis risk, whereas a balanced immune-inflammatory profile corresponds to a lower risk. Conversely, in adults without hyperlipidemia, systemic immune cell variations (within typical ranges) do not show a significant correlation with differences in periodontal health.

## Supplementary information

Supplementary Material

## Data Availability

The datasets analysed in our study are available in the National Center for Health Statistics (NCHS), https://www.cdc.gov/nchs/nhanes/index.html.
