# Supplementary material for: The association between systemic immune-inflammation index and periodontitis in adults with and without hyperlipidemia: a population-based study
Source: BDJ Open. 2026 Apr 1;12:29. doi: 10.1038/s41405-026-00422-3 (PMC13043662; doi:10.1038/s41405-026-00422-3)
Supplement: Supplementary file 1 — Supplementary Material [file 41405_2026_422_MOESM1_ESM.docx]

**Supplementary material**

**Supplementary Figure 1**. Relationship between SII and periodontitis stratified by hyperlipidemia in males (A), females (B), excluding individuals with malignancies (C) and using alternative periodontitis definitions (D).

**Supplementary Figure 2.** Relationship between SII and periodontitis using solely laboratory criteria to define hyperlipidemia.

**Supplementary Table 1.** Criteria for Periodontitis Classification.

**Supplementary Table 2.** Logistic regression analysis of the association between SII tertiles and periodontitis.


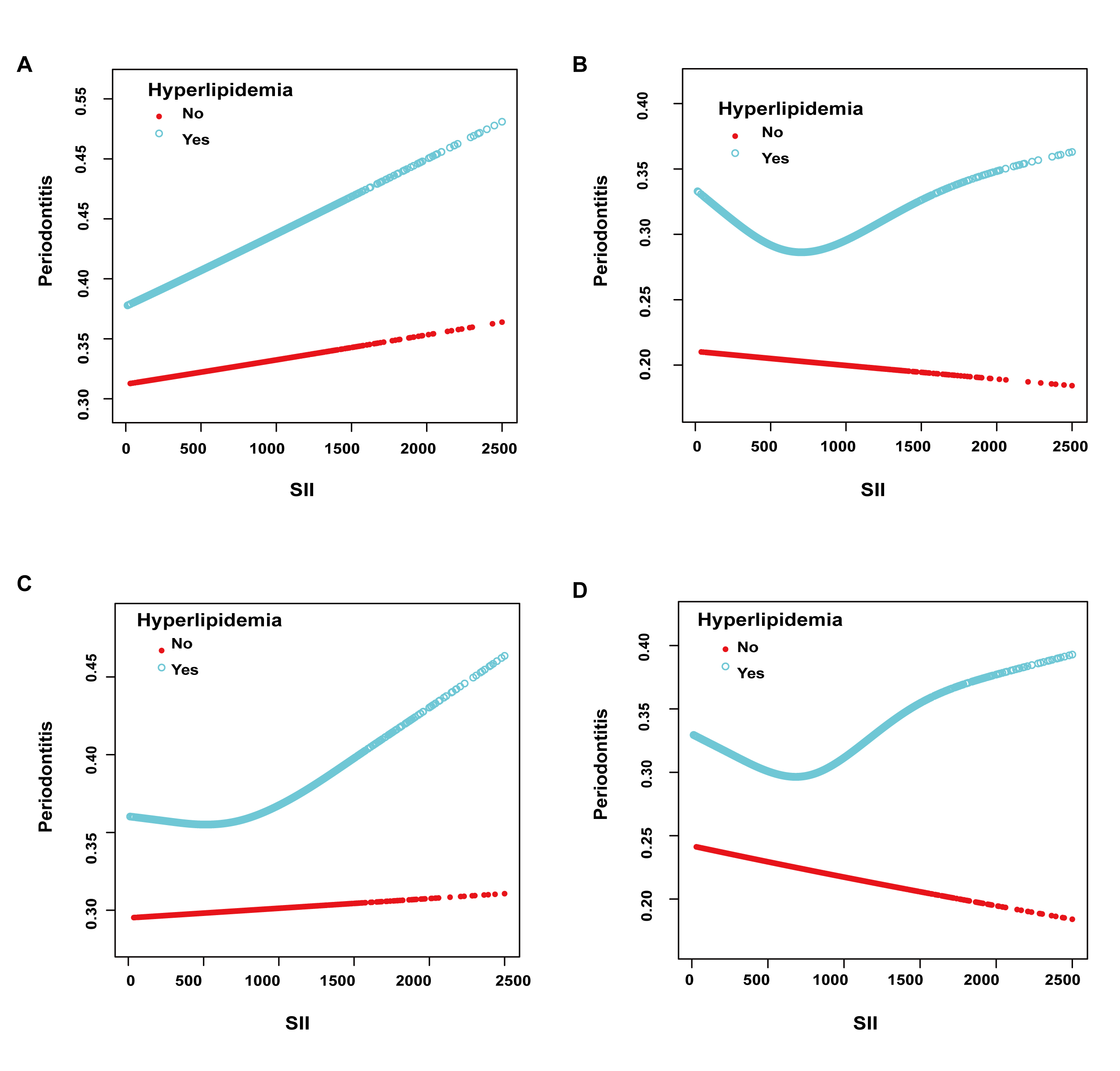


**Supplementary Figure 1**. Relationship between SII and periodontitis stratified by hyperlipidemia in males (A), females (B), excluding individuals with malignancies (C) and using alternative periodontitis definitions (D). Adjusted for age, gender, race, education, marital status, PIR, smoking status, alcohol consumption, total physical activity, hypertension, diabetes mellitus and BMI except for gender in (A) and (B).


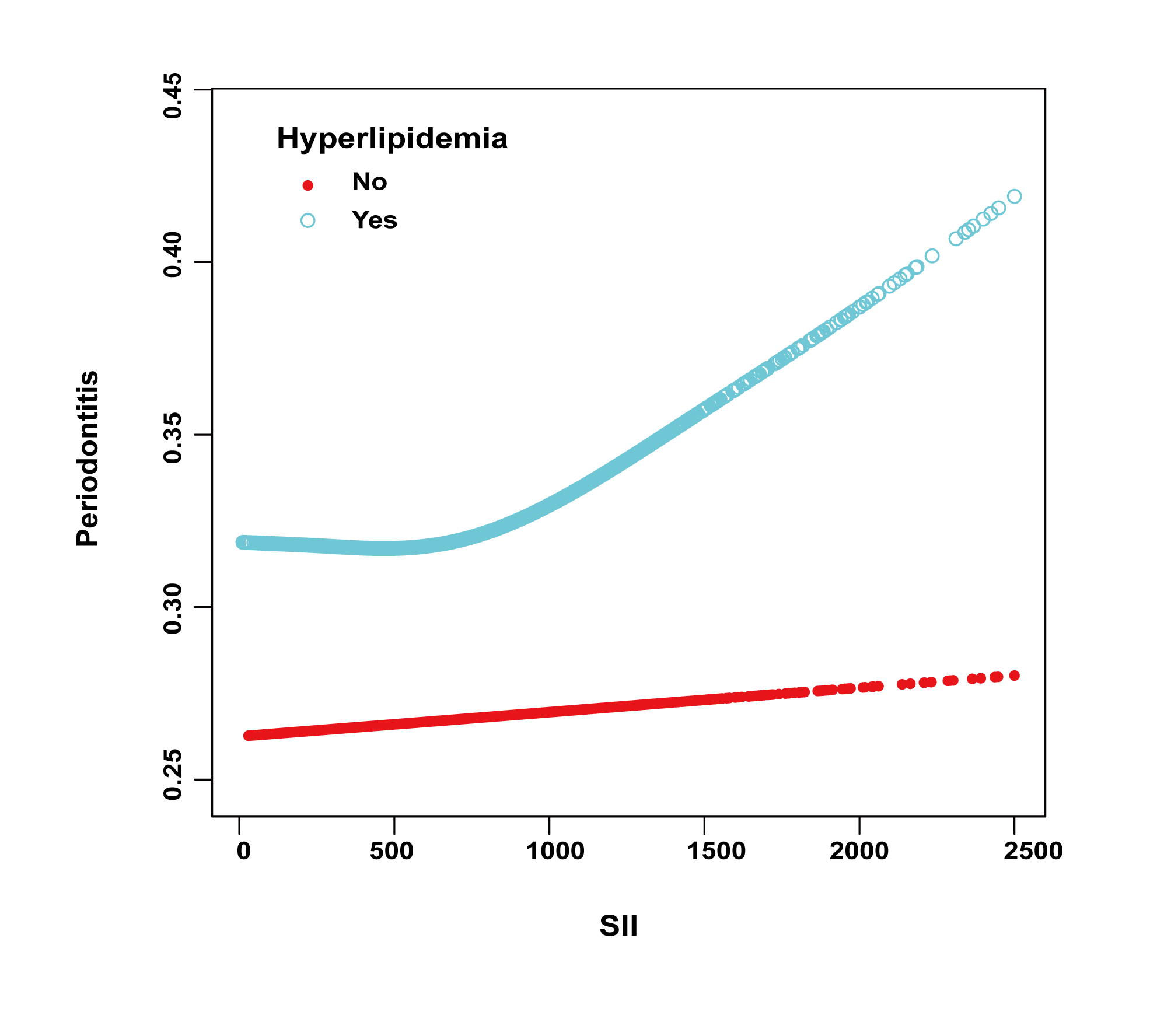


**Supplementary Figure 2.** Relationship between SII and periodontitis using solely laboratory criteria to define hyperlipidemia. Adjusted for age, gender, race, education, marital status, PIR, smoking status, alcohol consumption, total physical activity, hypertension, diabetes mellitus and BMI.

**Supplementary Table 1.** Criteria for Periodontitis Classification

| Periodontitis classification | Criteria |
| --- | --- |
| mild | ≥2 interproximal sites with CAL ≥ 3 mm and PPD ≥ 4mm, not on the same tooth |
| moderate | ≥2 interproximal sites with CAL ≥ 4 mm, not on the same tooth, or ≥ 2 interproximal sites with PPD ≥ 5 mm, not on the same tooth |
| severe | ≥2 interproximal sites with CAL ≥ 6 mm, not on the same tooth, and ≥1 interproximal site with PPD ≥ 5 mm |

Abbreviations: PPD probing pocket depth, CAL clinical attachment loss.

**Supplementary Table 2. Logistic regression of the association between SII and periodontitis.**

| Variable | Model I | | | |  | Model II | |  | Model III | |
| --- | --- | --- | --- | --- | --- | --- | --- | --- | --- | --- |
|  | **OR (95%CI)** | ***P*-value** | | |  | **OR (95%CI)** | ***P*-value** |  | **OR (95%CI)** | ***P*-value** |
| SII (per 500- unit increase) | 0.89 (0.85, 0.94) | | | <0.0001 |  | 1.05 (1.00, 1.10) | 0.06 |  | 1.06 (0.99, 1.14) | 0.09 |
| SII.T3 |  | | |  |  |  |  |  |  |  |
| T 1 | Ref. | | |  |  | Ref. |  |  | Ref. |  |
| T 2 | 0.81 (0.76, 0.87) | | | <0.0001 |  | 0.95 (0.88, 1.02) | 0.17 |  | 0.96 (0.87, 1.05) | 0.38 |
| T 3 | 0.76 (0.71, 0.82) | | | <0.0001 |  | 1.00 (0.92, 1.08) | 0.95 |  | 1.00 (0.91, 1.11) | 0.98 |
| *P* for trend |  | | <0.0001 | |  |  | 0.92 |  |  | 0.98 |

Model I was adjusted for none.

Model II was adjusted for age, gender, and ethnicity.

Model III was adjusted for age, gender, race, marital status, PIR, education, smoking status, alcohol consumption, physical activity and BMI.

Abbreviations: OR, odds ratio.
